# Exploratory Listening Through Background Music: Psychological Predictors of Everyday Use

**DOI:** 10.3390/bs16050770

**Published:** 2026-05-14

**Authors:** Guanqing Wu, Qian Zhang, Alexander Park, Kyung-Hyun Suh

**Affiliations:** 1 Conservatory of Music, Xianda College of Economics and Humanities, Shanghai International Studies University, Shanghai 200089, China; wuguanqingchris@sina.com (G.W.); 2411011@xdsisu.edu.cn (Q.Z.); 2 Department of Interdisciplinary Arts, Sahmyook University, Seoul 01795, Republic of Korea; hjpark@syu.ac.kr; 3 Department of Counseling Psychology, Sahmyook University, Seoul 01795, Republic of Korea

**Keywords:** background music, personality, temperament, self-efficacy, hardiness

## Abstract

This study investigated the psychological, temperamental, and experiential factors associated with background music use among Chinese adults and examined predictive models incorporating psychological variables, demographic characteristics, and music-related experiences. In total, 332 Chinese adults aged 18–65 years (*M* = 35.89, *SD* = 13.34) participated in the study. Background music use was analyzed using correlational analyses, stepwise regression, and decision tree modeling. Results indicated that extraversion, neuroticism, and self-efficacy were significantly associated with background music use. Among temperamental traits, fun seeking showed a small but significant negative relationship. Stepwise regression analysis revealed that extraversion accounted for the largest proportion of variance, followed by neuroticism and self-efficacy, with the overall model explaining a significant portion of the variance in background music use. The decision tree model further identified experiential and contextual variables, including formal music education, enjoyment of music classes in childhood, living arrangements, and music-related family experiences, as important factors differentiating usage patterns. These findings suggest that background music use among Chinese adults is shaped by arousal-related personality traits, self-regulatory resources, and early musical experience. The results provide useful implications for further research and offer foundational knowledge for understanding background music use in everyday contexts.

## 1. Introduction

The use of background and environmental music has been widely practiced across cultures, from ancient civilizations to the present day (Allen & Dawe, 2017; North & Hargreaves, 2004). In contemporary society, it has become an essential element in various life contexts such as studying, working, exercising, relaxing, and shopping (Ahlbom et al., 2023; Juslin & Sloboda, 2010). While ambient music has been discussed primarily in relation to shaping the atmosphere of physical spaces (Till, 2017), background music in everyday contexts refers more specifically to music that accompanies ongoing activities and supports individuals’ engagement with their tasks. Music extends beyond mere auditory stimuli; it serves multiple functions, including emotion regulation, concentration enhancement, and construction of social and environmental settings. In this sense, background music can be understood as a functional and self-directed resource that individuals use to manage their psychological states and task environments in daily life.

Background and environmental music have been shown to serve positive functions in individuals’ daily activities by facilitating emotion regulation and cognitive performance. Previous studies have suggested that environmental music plays a role in stabilizing or positively influencing emotional states, particularly in relation to stress reduction and mood regulation (Juslin & Sloboda, 2010; Saskovets et al., 2025). Moreover, music characterized by repetitive structures and low levels of stimulation can help mask external noise and promote psychological immersion, thereby contributing to sustained attention in learning and work settings (Kiss & Linnell, 2021; Lehmann & Seufert, 2017). Thus, background music may be understood not only as an incidental stimulus but also as a functional resource that individuals may use to regulate their environment and manage their psychological state.

Meanwhile, background and environmental music have been discussed as playing significant roles at the individual, social, and environmental levels. Environmental music used in commercial spaces and public settings, such as restaurants, department stores, and markets, has been shown to shape the atmosphere of a space and influence users’ behavior and attitudes by regulating their emotional experiences (Ahlbom et al., 2023; Malcman et al., 2024; Manzoor, 2024). For example, in-store music can affect consumers’ length of stay, purchase intentions, and evaluations of the environment, and these effects vary depending on the characteristics of the music and situational context (Biswas et al., 2019). Furthermore, environmental music may function as a medium that connects individuals with space and cultural contexts and is increasingly considered an important element that contributes to the quality of everyday environments in contemporary society (Allen & Dawe, 2017).

Although music is also used in commercial or public environments, the present study focuses specifically on individual differences in the self-directed use of background music in everyday personal contexts. Individual differences exist in how frequently such music is used, how it is utilized, and its effects (Carraturo et al., 2024). Some individuals actively incorporate background music into activities, such as studying, working, or relaxing, whereas others rarely use such music or even perceive it as a source of distraction (North & Hargreaves, 2008). Furthermore, even when exposed to the same musical environment, the magnitude and direction of the effects of music on attention, learning, and emotional responses may vary across individuals (Kiss & Linnell, 2021; Lehmann & Seufert, 2017). These differences have been reported to be closely associated with the characteristics of the music and psychological factors such as personality traits, cognitive resources, and motivational tendencies (Juslin & Sloboda, 2010). Therefore, to gain a more comprehensive understanding of the use and effects of background music, it is important to identify factors that are associated with and may help explain individual differences. Accordingly, this study sought to explore intrapersonal characteristics that might predict everyday background music use.

This study hypothesizes that individual personality traits are associated with background music use. Personality traits are closely associated with the degree to which individuals enjoy listening to music or playing musical instruments (K. Suh & Park, 2011; Zhao et al., 2025). For example, traits such as extraversion and openness have been found to be significantly associated with preferences for and engagement in musical activities, which may also be reflected in patterns of music use (Chamorro-Premuzic & Furnham, 2007; Chamorro-Premuzic et al., 2009). Previous studies have shown that the effects of background music may vary depending on individual personality characteristics (Deng et al., 2025; Lim et al., 2021). From the perspective of personality theory, the relationship between background music and cognitive performance has been suggested to interact with extraversion and arousal levels (Küssner, 2017). Moreover, findings that background music influences the perception of others’ personalities highlight the close relationship between background music use and personality (Lastinger, 2011). Collectively, these findings suggest the importance of exploring personality factors that may predict individual differences in personal background music use.

This study also examined the relationship between temperamental personality dispositions, namely the behavioral activation system (BAS) and behavioral inhibition system (BIS), and background music use. The BAS is a dopamine-based motivational system sensitive to rewards that promotes approach behavior and positive emotions, whereas the BIS is associated with threat sensitivity and behavioral inhibition, being closely associated with anxiety and other negative emotions (Gray, 1990; Urošević et al., 2008). Previous studies have suggested that individuals with higher BAS scores may be more sensitive to the rewarding aspects of music and, therefore, more likely to actively engage in musical activities (Loxton et al., 2016). However, it has also been argued that a high BAS score, which is associated with impulsivity, may be negatively associated with sustained musical activities that require continuous practice and long-term effort (Smillie et al., 2006; Zhao et al., 2025). Therefore, we assumed that individual differences in background music use may vary depending on levels of BAS and BIS sensitivity.

This study assumes that self-efficacy is associated with background music use. Self-efficacy, a concept proposed by Bandura (1982), refers to an individual’s belief in their ability to successfully perform specific tasks and achieve desired goals. Previous studies have demonstrated that self-efficacy is associated not only with achievement in musical activities but also with music preferences and motivation (Chen, 2024; Zelenak, 2024). Although self-efficacy may influence music use, music use may also contribute to self-efficacy enhancement. For example, a study conducted in elementary school language classrooms found that the use of background music was associated with increased self-efficacy (Van Horn, 2020). Therefore, we examined whether self-efficacy functioned as an intrapersonal psychological factor associated with background music use.

Hardiness may also be associated with background music use. According to Kobasa (1979a), psychological hardiness refers to a disposition characterized by a commitment to life, a sense of control over events, and a tendency to perceive challenges as opportunities for growth. In line with this conceptualization, Park and Suh (2023) have reported that hardiness is positively associated with listening to music for negative emotion regulation and life satisfaction, suggesting that individuals with high hardiness may actively use music as a coping resource. Background music has been reported to exert positive influences on individuals’ approaches to life and motivation as well as on emotion regulation, cognitive engagement, and levels of arousal (Nair & Patil, 2026). Moreover, previous research has indicated that the type of background sound, such as relaxing or stimulating music, can influence cognitive task performance and error rates, implying that individuals’ responses to background music may depend on their ability to manage environmental stimuli (Nadon et al., 2021). Therefore, we hypothesize that hardiness is associated with individual differences in background music use.

This study aimed to examine how personality traits, temperament (BAS/BIS), self-efficacy, and hardiness were associated with individuals’ tendency to use background music and to explore factors associated with background music use among Chinese adults. Specifically, this study addresses the following research questions: First, are there significant relationships among the Big Five personality traits, BAS/BIS, self-efficacy, hardiness, and the tendency to use background music among Chinese adults? Second, what variables are identified in the stepwise regression model as exploratory predictors of background music use? Third, what patterns can be identified using a decision tree model to describe variations in background music use?

Because background music is widely embedded in daily and social contexts, understanding who tends to use it is theoretically and practically important. Identifying psychological predictors of background music use can clarify individual differences in music use patterns and support personalized and effective applications of music in educational, occupational, and public settings. This perspective is also consistent with recent views of music listening as exploratory behavior involving active regulation of attention, arousal, and engagement with the sonic environment (Reybrouck et al., 2024).

## 2. Materials and Methods

### 2.1. Participants

In total, 332 Chinese adults participated in this study. The participants’ age ranged from 18 to 65 years, with a mean age of 35.89 years (SD = 13.34).

### 2.2. Participants’ Characteristics

Of the participants, 148 (44.6%) were male and 184 (55.4%) were female (Table 1). Of them, 143 were in their 20s or younger (43.1%), 65 in their 30s (19.6%), 54 in their 40s (16.3%), 43 in their 50s (13.0%), and 27 in their 60s (8.1%). Eighty (24.1%) participants reported living alone.

Regarding musical background, 44 (13.3%) participants were currently majoring in music or had majored in music. Additionally, 37 (11.1%) had at least one parent who majored in music, and 54 (16.3%) had siblings who were or had been music majors. When asked about childhood experiences with music, 104 (31.3%) participants reported enjoying music classes during childhood, whereas 97 (29.2%) had received music education.

### 2.3. Data Collection

Data were collected by Shanghai Shangzi Market Consulting Co., Ltd., a professional online research agency in Shanghai, China. Participants were recruited from the agency’s nationwide panel of registered mobile users and completed the survey online on a voluntary basis. Eligible participants were Chinese adults aged between 18 and 65 years, with individuals outside this age range excluded. The survey was structured to require responses to all items before submission, resulting in no missing data in the final dataset, which is typical in structured online panel surveys. Upon completion, participants received a small incentive in the form of reward points equivalent to approximately USD 2, following the agency’s standard compensation procedures.

Prior to data collection, ethical approval was granted by the Institutional Review Board (IRB). Informed consent was obtained electronically from all participants, and every effort was made to ensure that the data collection process adhered to ethical standards. After providing consent to participate in the online survey, participants were informed that they could withdraw from the study at any time if they experienced any discomfort while completing the survey. They were also notified that their responses would be used solely for research purposes and that all data would be securely stored on an encrypted computer for three years, after which it would be permanently deleted.

### 2.4. Instruments

#### 2.4.1. Background Music Use

This study used the background use subscale of the Use of Music Inventory (UMI) developed by Chamorro-Premuzic and Furnham (2007) to assess the participants’ use of music as a background in daily life. This subscale consists of five items, one of which is reverse scored. Example items include “I enjoy listening to music while I work” and “Music is very distracting so whenever I study, I need to have silence” (reverse scored). Participants responded on a five-point Likert scale ranging from 1 (strongly disagree) to 5 (strongly agree). Although the internal consistency of the background use subscale in this study was relatively modest (Cronbach’s alpha = 0.62), a previous study using Chinese adult samples has reported higher reliability (e.g., α = 0.84; Q. Zhang & Suh, 2026). The lower reliability observed in this study may be attributable to the small number of items and the inclusion of a reverse-scored item, both of which are known to attenuate alpha coefficients (Cortina, 1993), and thus can be considered acceptable.

#### 2.4.2. Personality Traits

The participants’ personality traits were assessed using the Chinese Big Five Personality Inventory-15 (CBF-PI-15; X. Zhang et al., 2019), which is based on the Big Five personality framework and evaluates five core dimensions: neuroticism, extraversion, conscientiousness, openness, and agreeableness. The inventory consisted of 15 items, including two reverse-scored items. Each item is rated on a six-point Likert scale ranging from 1 (strongly disagree) to 6 (strongly agree). The scale demonstrated satisfactory reliability and validity in previous studies (X. Zhang et al., 2019). Although certain subscales yield relatively low internal consistency coefficients in the present study, this is deemed acceptable given that Cronbach’s alpha is known to be influenced by the number of items in each subscale. In this study, Cronbach’s alphas for the five dimensions were as follows: 0.82 for neuroticism, 0.87 for extraversion, 0.76 for conscientiousness, 0.62 for openness, and 0.76 for agreeableness.

#### 2.4.3. BAS/BIS

The BAS and BIS sensitivities were assessed using the BAS/BIS Scale developed by Carver and White (1994). This study used the Chinese version of the scale validated by Che et al. (2020). The scale comprises 18 items and three BAS subscales: reward responsiveness (four items), drive (four items), and fun seeking (five items), which reflect behavioral activation and sensitivity to rewards. The BIS subscale consists of five items that measure behavioral inhibition and sensitivity to threats. All items were rated on a four-point Likert scale ranging from 1 (strongly agree) to 4 (strongly disagree). In this study, all items were reverse scored such that higher scores indicated greater BAS and BIS sensitivities. Cronbach’s alpha for the BAS subscales ranged from 0.64 to 0.76, and the alpha for the BIS subscale was 0.79.

#### 2.4.4. Self-Efficacy

Self-efficacy was assessed using the Chinese version of the General Self-Efficacy Scale developed by J. X. Zhang and Schwarzer (1995). This instrument comprises 10 items designed to measure individuals’ beliefs in their ability to cope with and overcome challenges in daily life. Responses were recorded on a four-point Likert scale ranging from 1 (not at all true) to 4 (exactly true). In this study, the scale demonstrated strong internal consistency with a Cronbach’s alpha of 0.87.

#### 2.4.5. Hardiness

Psychological hardiness was measured using a brief measure developed by K. H. Suh (2022). The scale included 12 items distributed across three subscales: commitment (four items), self-directedness (four items), and tenacity (four items). Each item is rated on a six-point Likert scale ranging from 1 (not at all true) to 6 (very true), with higher scores indicating greater psychological hardiness. This scale was translated and applied to Chinese samples following an appropriate translation procedure (Ouyang et al., 2024), supporting its linguistic and contextual suitability. In addition, a previous study with Chinese adult samples has reported satisfactory internal consistency for this measure (Zhao et al., 2025), indicating its reliability in a similar population. In this study, Cronbach’s alphas were 0.84 for commitment, 0.80 for self-directedness, 0.72 for tenacity, and 0.92 for the overall scale.

### 2.5. Statistical Analysis

All data analyses were conducted using IBM SPSS Statistics for Windows version 26. Prior to the parametric statistical analyses, the normality of the psychological variables was examined by assessing skewness and kurtosis. Based on the results, correlation, multiple regression, and stepwise regression analyses were performed using parametric statistical methods. In addition, decision tree analysis was conducted as a non-parametric approach to explore classification patterns in background music use.

The Chi-square Automatic Interaction Detection (CHAID) algorithm was used for decision tree analysis. Originally developed by Kass (1980), CHAID performs multi-way splits based on the chi-square (χ^2^) statistic for categorical dependent variables and the F-statistic from analysis of variance for continuous dependent variables. In this study, the total score was used as the target variable. Because this variable was continuous, the splitting procedure was based on the F-statistic derived from analysis of variance. The maximum depth of the tree was limited to three levels, and the minimum number of cases required for the parent and child nodes was set to 30 and 10, respectively.

## 3. Results

### 3.1. Relationships Among Personality, BAS/BIS, Self-Efficacy, Hardiness, and Background Music Use

Table 2 shows the results of the correlation analysis among the Big Five personality traits, BAS/BIS, self-efficacy, hardiness, and background music use in a sample of 332 Chinese adults. The absolute values of skewness and kurtosis for all variables were within ±2.0, indicating no significant deviation from normality and justifying the use of parametric statistical methods (Kline, 2005).

The analysis revealed that extraversion (*r* = 0.37, *p* < 0.001), conscientiousness (*r* = 0.17, *p* < 0.01), and agreeableness (*r* = 0.16, *p* < 0.01) positively correlated with background music use. By contrast, neuroticism (*r* = 0.25, *p* < 0.001) and openness (*r* = −0.06, n.s.) were not significantly correlated.

Regarding temperament, neither the BAS (*r* = −0.03, n.s.) nor the BIS (*r* = −0.02, n.s.) showed a significant correlation with background music use. Among the BAS subcomponents, only fun seeking demonstrated a small but significant negative correlation (*r* = −0.13, *p* < 0.05). By contrast, reward responsiveness (*r* = 0.08, n.s.) and drive (*r* = −0.03, n.s.) were not significantly associated with background music use.

In terms of psychological resources, self-efficacy showed a significant positive correlation with background music use (*r* = 0.27, *p* < 0.001), whereas hardiness was also positively correlated but with a smaller effect (*r* = 0.16, *p* < 0.01). Regarding the subcomponents of hardiness, commitment (*r* = 0.19, *p* < 0.001) and self-directedness (*r* = 0.13, *p* < 0.05) were significantly positively associated with background music use, whereas tenacity was not significantly correlated (*r* = 0.10, n.s.).

### 3.2. Exploratory Models for Background Music Use

This study explored potential factors associated with background music use among Chinese adults. First, multiple regression analysis was conducted using the Big Five personality traits.

Table 3 shows the results of the multiple regression analysis examining the extent to which the Big Five personality traits predict background music use. The overall model was statistically significant, *F*(5, 326) = 14.66, *p* < 0.001, and explained approximately 18.4% of the variance in background music use (*R*^2^ = 0.184, *adj. R*^2^ = 0.174).

Among the five personality traits, extraversion (*β* = 0.325, *t* = 5.89, *p* < 0.001) and neuroticism (*β* = 0.232, *t* = 4.25, *p* < 0.001) were significant positive predictors of background music use. This suggests that individuals with higher levels of extraversion and neuroticism are more likely to use background music. However, conscientiousness, openness, and agreeableness did not significantly predict background music use in the model.

An exploratory stepwise regression analysis is presented in Table 4. Extraversion was entered at the first step and accounted for a significant proportion of variance in background music use (*β* = 0.365, *t* = 7.13, *p* < 0.001), explaining 13.4% of the variance (*R*^2^ = 0.134, *adj. R*^2^ = 0.131, *F* = 50.86, *p* < 0.001). The inclusion of neuroticism in the second step significantly increased the explained variance by 4.3% (Δ*R*^2^ = 0.043), with neuroticism emerging as a significant predictor (*β* = 0.214, *t* = 4.26, *p* < 0.001), resulting in a cumulative *R*^2^ of 0.179 (*adj. R*^2^ = 0.174, *F* = 35.84, *p* < 0.001). Finally, self-efficacy was added to the model, contributing an additional 5.2% to the explained variance (Δ*R*^2^ = 0.052), and was also a significant predictor (*β* = 0.256, *t* = 4.86, *p* < 0.001). The final model explained 23.4% of the variance in background music use (*R*^2^ = 0.234, *adj. R*^2^ = 0.227, *F* = 33.40, *p* < 0.001).

To explore decision-tree patterns of background music use among Chinese adults, variables including psychological, demographic, experiential, and contextual factors were entered as potential predictors.

**Figure 1:**
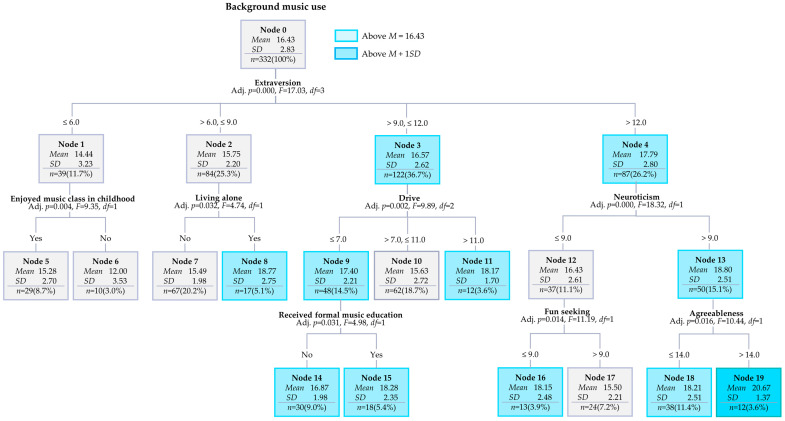
Decision tree model of background music use among Chinese adults.

Results revealed that the total number of nodes was 20, the number of terminal nodes was 12, and the number of depths was 3 (Figure 1). The risk estimate was 5.49 (*SE* = 0.51), and the average risk estimate of the 10-fold cross-validation was 7.60 (*SE* = 0.69), indicating differences in the margin of error.

The average background music usage by the root nodes was 16.43. Eleven nodes (Nodes 3, 4, 8, 9, 11, 13, 14, 15, 16, 18, and 19) exceeded this average, and Chinese adults belonging to these nodes scored higher on background music use (Figure 1). The order of gain nodes was 19 (3.6%), 15 (5.4%), 18 (11.4%), 11 (3.6%), 16 (3.9%), 14 (9.0%), 8 (5.1%), 10 (18.7%), 17 (7.2%), 7 (20.2%), 5 (8.7%), and 6 (3.0%; Table 5).

The first criterion used to classify background music use was extraversion (Figure 1). Participants with extraversion scores of six or lower were grouped into Node 1, and their average background music use score was 14.44. Among them, those who reported having enjoyed music classes in childhood (Node 5) had a higher average score of 15.28, whereas those who did not enjoy such classes (Node 6) had a lower average score of 12.00.

Participants with extraversion scores between six and nine were classified into Node 2, with an average score of 15.75. In this group, the participants who lived with others (Node 7) had an average score of 15.49, whereas those who lived alone (Node 8) had an average score of 18.77.

Those whose extraversion scores ranged between nine and 12 were placed in Node 3, with an average score of 16.57. This group was further divided according to drive scores. Participants with drive scores of 7 or lower (Node 9) had an average of 17.40. Those with drive scores between 7 and 11 (Node 10) had an average score of 15.63, and participants with drive scores higher than 11 (Node 11) had an average score of 18.17.

Node 9 was further subdivided according to whether participants had received formal music education. Among those who had not received formal music education (Node 14), the mean background music usage score was 16.87. By contrast, participants who had received formal music education (Node 15) had a higher mean score of 18.28, indicating that formal music education was associated with greater background music use in this subgroup.

Participants with extraversion scores higher than 12 were categorized into Node 4, which had an average background music usage score of 17.79. This group was then split according to neuroticism scores. Participants with neuroticism scores of 9 or lower (Node 12) had an average score of 16.43, whereas those with neuroticism scores higher than 9 (Node 13) had an average score of 18.80.

Participants in Node 12 were further classified according to their fun-seeking scores. Those whose fun-seeking scores were 9 or lower (Node 16) had an average score of 18.15. By contrast, those with scores higher than 9 (Node 17) had an average score of 15.50.

Participants in Node 13, with high neuroticism scores, were further divided based on their agreeableness scores. Those whose agreeableness scores were 14 or lower (Node 18) had an average background music usage score of 18.28. Those whose agreeableness scores were higher than 14 (Node 19) recorded the highest average score in the entire decision tree at 20.67.

## 4. Discussion

This study explored factors associated with background music use among Chinese adults with the aim of contributing foundational knowledge for future research and providing preliminary insights into music-related practices in everyday contexts. Psychological variables, including personality traits, BAS/BIS sensitivity, self-efficacy, and hardiness, which were theoretically and empirically associated with background music use, were analyzed using a stepwise regression model. In addition, demographic characteristics, including categorical variables that might be associated with patterns of music use, were incorporated into a decision tree model to explore patterns of background music use. The implications of these findings are as follows:

The positive association between extraversion and background music use can be interpreted based on Eysenck’s arousal theory, which proposes that extraverts, characterized by a relatively lower baseline cortical arousal, seek additional external stimulation to achieve an optimal arousal level (Eysenck, 1967). Building on this framework, Küssner (2017) reviewed evidence suggesting that the cognitive effects of background music varied depending on extraversion, with extraverts often benefiting more from auditory stimulation. Moreover, previous empirical studies have consistently shown that extraversion is associated with more frequent and functional uses of music, such as mood regulation and accompanying activities (Chamorro-Premuzic & Furnham, 2007; Chamorro-Premuzic et al., 2009). The positive correlation between conscientiousness and agreeableness may reflect different psychological mechanisms. Conscientious individuals may deliberately use background music as a strategy to organize their environment and sustain task engagement, which is consistent with self-regulatory perspectives on environmental management (North & Hargreaves, 2008). Conversely, agreeableness may be associated with a preference for maintaining emotionally pleasant and harmonious surroundings facilitated by background music.

The positive relationship between neuroticism and background music use suggests that individuals with higher emotional reactivity may be more likely to use background music as a means of managing their affective states. Individuals high in neuroticism tend to experience greater emotional fluctuation and psychological tension, and music may function as an accessible resource for mood regulation in everyday contexts. This interpretation is consistent with previous findings that neuroticism is associated with emotion-oriented uses of music, including coping and affect regulation (Chamorro-Premuzic & Furnham, 2007). However, given the correlational nature of the present study, this interpretation should be considered tentative, and further research is needed to clarify the causal role of emotional reactivity in background music use.

The non-significant association with openness is noteworthy. Although openness has been strongly linked to aesthetic sensitivity and diverse music preferences (Chamorro-Premuzic et al., 2009), it may be more relevant to intentional and immersive music engagement than to incidental background use. Collectively, these findings suggest that background music use is more closely associated with stimulation seeking and self-regulatory tendencies than with aesthetic curiosity alone.

The regression analyses indicated that among the Big Five traits, only extraversion and neuroticism were independently associated with background music use, with extraversion emerging as the strongest predictor. This finding is consistent with Eysenck’s arousal theory (Eysenck, 1963, 1967), which suggests that extraverts seek additional external stimulation, and background music may help optimize arousal levels. Consistent with previous studies (Chamorro-Premuzic & Furnham, 2007; Rentfrow & Gosling, 2003), extraversion appeared to be an important dispositional basis for everyday music use. Importantly, decision tree analysis further supported this pattern, as extraversion functioned as the primary splitting variable, indicating its hierarchical importance in differentiating the levels of background music use. Neuroticism also contributed to the unique variance in the regression model and emerged as a secondary splitting factor in the higher extraversion groups in the decision tree, suggesting that emotional reactivity also shaped music use patterns. Overall, converging evidence from both analytical approaches indicates that background music use appears to be largely shaped by arousal- and emotion-related personality dimensions. The stepwise regression analysis should be interpreted as exploratory, as it was used to identify potential predictors rather than to establish a stable predictive model.

Neither the BIS nor the overall BAS was significantly associated with background music use, suggesting that general threat sensitivity or broad approach motivation may not be directly related to this behavior. Only the fun-seeking subcomponent of the BAS showed a small but significant negative association. Fun seeking reflects a tendency toward a spontaneous approach behavior and the pursuit of immediate enjoyment and novel stimulation (Carver & White, 1994). This negative association suggests that individuals who are more strongly oriented toward immediate pleasure and novelty may be less likely to use background music in a sustained or functional manner during everyday activities. In other words, background music use in daily life may reflect not only hedonic motivation but also a degree of task-related regulation and environmental structuring, which may not align with a more impulsive fun-seeking tendency. However, given the relatively small effect size, this finding should be interpreted with caution, as temperamental approach tendencies may reflect context-specific patterns rather than a central or general determinant of background music use.

The positive association between self-efficacy and background music use, along with its additional contribution in the stepwise regression model, suggests that background music use is associated with personality traits and individuals’ perceived competence in regulating their behavior and environment. According to Bandura (1982, 1997), self-efficacy facilitates proactive self-regulation and strategic actions. Therefore, individuals with higher self-efficacy may intentionally use background music to enhance their concentration or manage their mood during daily tasks. In support of this interpretation, a recent meta-analysis by Zelenak (2024) demonstrated that self-efficacy was positively associated with engagement and performance in music-related contexts, suggesting that confidence beliefs systematically influenced individuals’ use of musical activities. Furthermore, self-efficacy remained significant beyond extraversion and neuroticism, indicating its unique role as a psychological resource associated with background music use.

The positive associations of commitment and self-directedness with background music use suggest that individuals who are actively engaged in their activities and perceive greater personal control may be more likely to use background music as a functional resource. According to hardiness theory (Kobasa, 1979b; Maddi, 2002), commitment reflects meaningful involvement in life tasks, whereas control is generally understood as the belief that one can influence outcomes through one’s own efforts. Self-directedness captures a sense of internal control and agency. Therefore, individuals with higher levels of these traits may be more likely to structure their environments, including through the use of background music, to sustain engagement, regulate mood, and manage daily demands. Previous studies have also associated hardiness with adaptive coping and proactive stress management (Eschleman et al., 2010), which is consistent with this interpretation. By contrast, tenacity was not significantly associated, suggesting that persistence without perceived control or active engagement might be less relevant to background music use.

The decision tree analysis further suggested that, beyond internal psychological variables, experiential and contextual factors, such as formal music education, living alone, and enjoyment of music classes in childhood, were associated with differences in patterns of background music use. Individuals who received formal music education tended to show higher levels of background music use in certain subgroups, suggesting that structured musical training may be associated with greater familiarity with and more intentional use of music in everyday life. Similarly, living alone was associated with higher background music use, implying that music might serve as a psychological and situational resource in solitary contexts, supporting mood regulation and engagement during everyday activities (Schäfer & Eerola, 2020). In addition, those who reported enjoying music classes in childhood tended to show greater background music use, indicating that early positive musical experiences may be associated with greater engagement with music (McPherson & Welch, 2012). Together, these findings suggest that background music use may be influenced not only by internal psychological characteristics but also by developmental experiences and current living contexts.

This study has several limitations that should be considered when interpreting the findings. First, although data were collected from Chinese adults across various regions through an online survey, the sample could not be considered fully representative of the broader population because online recruitment might introduce self-selection and accessibility biases. Second, the correlational design limits causal interpretations. Although the discussion is grounded in previous theoretical and empirical studies, causal conclusions cannot be drawn, and such relationships would require experimental or longitudinal approaches. Third, although decision tree analysis offers advantages in modeling hierarchical relationships and handling mixed data types, the use of SPSS-based procedures may involve limitations related to model stability and generalizability.

In addition, the use of stepwise regression may involve limitations related to model stability and variable selection. Therefore, the findings from this analysis should be interpreted with caution, and future studies are encouraged to employ theory-driven approaches, such as hierarchical regression, to validate these results. Finally, background music use was measured using a brief subscale of the Use of Music Inventory (Chamorro-Premuzic & Furnham, 2007), which might not have comprehensively captured the multifaceted nature of background music use in everyday personal contexts. In this study, the internal consistency of this subscale was relatively modest, although a previous study with Chinese adult samples has reported higher reliability (e.g., Q. Zhang & Suh, 2026). This discrepancy may be attributable to the limited number of items and the inclusion of a reverse-scored item, which can attenuate reliability estimates. Future research could benefit from developing a more reliable and psychometrically well-validated instrument that broadly assesses background music use across diverse everyday contexts. Despite these limitations, the present study provides meaningful insights into individual differences in background music use and offers a foundation for future research and for developing hypotheses about how music may function in everyday contexts. The present findings may also contribute to discussions of exploratory listening, as Reybrouck et al. (2024) suggested that music listening involves active interactions with one’s sonic environment. In this regard, background music use may reflect everyday regulation of attention, arousal, and environmental engagement.

## 5. Conclusions

This study suggested that background music use among Chinese adults is associated with a combination of personality traits, temperamental tendencies, psychological resources, and musical experience. Extraversion emerged as the most prominent predictor, with neuroticism and self-efficacy contributing to unique explanatory power. Fun seeking showed a small but meaningful negative association with background music use, and specific components of hardiness, particularly commitment and self-directedness, also showed meaningful associations. The decision tree analysis further revealed that experiential and contextual variables, including formal music education, enjoyment of music classes in childhood, living arrangements, and music-related family experiences, played important roles in differentiating the patterns of background music use. Overall, these findings suggest that background music use reflects both dispositional characteristics and developmental and environmental influences. The results provide meaningful implications for future research and offer foundational knowledge for understanding patterns of background music use in everyday life and for informing future studies in applied and real-world contexts.

## Figures and Tables

**Table 1:** Characteristics of participants (*N* = 332).

Variables		Frequency	Percent (%)
Sex	Male	148	44.6
	Female	184	55.4
Age	20s or less	143	43.1
	30s	65	19.6
	40s	54	16.3
	50s	43	13.0
	60s	27	8.1
Housing type	Living alone	80	24.1
	Living with other(s)	252	75.9
Majoring or majored in music?	Yes	44	13.3
	No	288	86.7
Did your parents major in music?	Yes	37	11.1
	No	295	88.9
Are or were any of your siblings music majors?	Yes	54	16.3
	No	278	83.7
Enjoyed music classes in childhood?	Yes	104	31.3
	No	228	68.7
Have you ever received any form of music education?	Yes	97	29.2
	No	235	70.8

**Table 2:** Correlational matrix for the Big-5 personality, BAS/BIS, self-efficacy, hardiness, and background music use (*N* = 332).

Variables	1	2	3	4	5	6	6-1	6-2	6-3	7	8	9	9-1	9-2	9-3	10
1. Neuroticism	1															
2. Extraversion	0.09	1														
3. Conscientiousness	0.07	0.38 ***	1													
4. Openness	−0.37 ***	0.12 *	0.04	1												
5. Agreeableness	−0.01	0.31 ***	0.61 ***	0.08	1											
6. BAS	0.07	−0.19 ***	−0.31 ***	−0.08	0.30 ***	1										
6-1. Reward responsiveness	0.08	0.01	−0.29 ***	−0.02	0.27 ***	0.84 ***	1									
6-2. Drive	0.11 *	−0.19 ***	−0.31 ***	−0.10	0.28 ***	0.86 ***	0.60 ***	1								
6-3. Fun-seeking	−0.01	−0.30 ***	−0.20 ***	−0.08	−0.21 ***	0.85 ***	0.54 ***	0.63 ***	1							
7. BIS	0.26 ***	0.13 *	−0.08	0.26 ***	−0.04	0.34 ***	0.32 ***	0.21 ***	0.31 ***	1						
8. Self-efficacy	0.24 ***	0.30 ***	0.26 ***	0.13 *	0.28 ***	−0.03	0.05	−0.08	−0.05	0.16 **	1					
9. Hardiness	−0.12 *	0.40 ***	0.59 ***	0.10	0.59 ***	−0.35 ***	−0.27 ***	−0.39 ***	−0.22 ***	−0.01	0.42 ***	1				
9-1. Commitment	−0.20 ***	0.44 ***	0.50 ***	0.22 ***	0.53 ***	−0.27 ***	−0.15 **	−0.34 ***	−0.21 ***	−0.12 *	0.43 ***	0.88 ***	1			
9-2. Self-directedness	−0.04	0.34 ***	0.55 ***	0.02	0.53 ***	−0.35 ***	−0.32 ***	−0.37 ***	−0.20 ***	−0.07	0.34 ***	0.91 ***	0.68 ***	1		
9-3. Tenacity	−0.08	0.30 ***	0.53 ***	0.04	0.53 ***	−0.32 ***	−0.28 ***	−0.35 ***	−0.19 ***	−0.07	0.35 ***	0.91 ***	0.67 ***	0.78 ***	1	
10. Background music use	0.25 ***	0.37 ***	0.17 **	−0.01	0.16 **	−0.03	0.08	−0.04	−0.13 *	−0.02	0.27 ***	0.16 **	0.19 ***	0.13 *	0.10	1
*M*	9.83	10.56	12.40	10.38	12.31	27.55	7.74	8.35	11.45	11.42	27.02	50.42	16.28	17.06	17.08	16.43
*SD*	2.84	3.14	2.57	2.33	2.40	5.91	2.42	2.19	2.35	2.35	5.15	9.04	3.55	3.25	3.28	2.83
Skewness	−0.23	−0.21	−0.20	0.08	−0.43	0.83	1.02	0.63	0.21	0.21	−0.17	−0.24	−0.10	−0.23	−0.36	−0.10
Kurtosis	−0.25	−0.23	0.13	0.79	0.61	0.89	0.80	0.23	0.29	−0.48	−0.32	−0.04	−0.39	−0.01	0.49	0.66

* *p* < 0.05, ** *p* < 0.01, *** *p* < 0.001.

**Table 3:** Results of multiple regression analysis predicting background music use from the Big Five personality traits.

Variables	*B*	*SE*	*β*	*t*	*F*	*R* ^2^	*Adj. R* ^2^
Constant	9.330	1.231		7.96 ***	14.66 ***	0.184	0.171
Neuroticism	0.231	0.054	0.232	4.25 ***			
Extraversion	0.293	0.050	0.325	5.89 ***			
Conscientiousness	−0.017	0.066	−0.015	−0.23			
Openness	0.048	0.072	0.040	0.73			
Agreeableness	0.079	0.075	0.067	1.05			

*** *p* < 0.001.

**Table 4:** Results of the stepwise regression analysis of background music use.

Variables	*B*	*SE*	*β*	*t*	∆*R*^2^	*R* ^2^	*Adj. R* ^2^	*F*
Extraversion	0.330	0.046	0.365	7.13 ***	0.134	0.134	0.131	50.86 ***
Neuroticism	0.213	0.050	0.214	4.26 ***	0.043	0.179	0.174	35.84 ***
Self-efficacy	0.141	0.029	0.256	4.86 ***	0.052	0.234	0.227	33.40 ***

*** *p* < 0.001.

**Table 5:** Gain summary for nodes.

Nodes	*N*	*%*	*M*
19	12	3.6	20.67
15	18	5.4	18.28
18	38	11.4	18.21
11	12	3.6	18.17
16	13	3.9	18.15
14	30	9.0	16.87
8	17	5.1	16.76
10	62	18.7	15.63
17	24	7.2	15.50
7	67	20.2	15.49
5	29	8.7	15.28
6	10	3.0	12.00

Growing method: CHAID.

## Data Availability

The datasets used and analyzed in this study can be obtained from the corresponding author upon reasonable request.

## Abbreviations

BAS	behavioral activation system
BIS	behavioral inhibition system
SNSs	social network sites
CBF-PI-15	Chinese Big Five Personality Inventory-15
SPSS	Statistical Package for the Social Sciences
CHAID	chi-square automatic interaction detection
